# Transcriptome Profiling of Human Pre-Implantation Development

**DOI:** 10.1371/journal.pone.0007844

**Published:** 2009-11-16

**Authors:** Pu Zhang, Marco Zucchelli, Sara Bruce, Fredwell Hambiliki, Anneli Stavreus-Evers, Lev Levkov, Heli Skottman, Erja Kerkelä, Juha Kere, Outi Hovatta

**Affiliations:** 1 Department of Bioscience and Nutrition, Karolinska Institutet, Stockholm, Sweden; 2 Division of Obstetrics and Gynaecology, Department for Clinical Science, Intervention and Technology, Karolinska University Hospital Huddinge, Stockholm, Sweden; 3 Department of Women's and Children's Health, Uppsala Academic hospital, Uppsala, Sweden; 4 REGEA, Institute for Regenerative Medicine, University of Tampere and Tampere University Hospital, Tampere, Finland; 5 Department of Medical Genetics, University of Helsinki, and Folkhälsan Institute of Genetics, Helsinki, Finland; Ecole Normale Supérieure de Lyon, France

## Abstract

**Background:**

Preimplantation development is a crucial step in early human development. However, the molecular basis of human preimplantation development is not well known.

**Methodology:**

By applying microarray on 397 human oocytes and embryos at six developmental stages, we studied the transcription dynamics during human preimplantation development.

**Principal Findings:**

We found that the preimplantation development consisted of two main transitions: from metaphase-II oocyte to 4-cell embryo where mainly the maternal genes were expressed, and from 8-cell embryo to blastocyst with down-regulation of the maternal genes and up-regulation of embryonic genes. Human preimplantation development proved relatively autonomous. Genes predominantly expressed in oocytes and embryos are well conserved during evolution.

**Significance:**

Our database and findings provide fundamental resources for understanding the genetic network controlling early human development.

## Introduction

Preimplantation development is the first step of individual life in mammals. It includes a series of important developmental events: final maturation of the oocyte, fertilization, oocyte to zygote transition, cell proliferation and differentiation, and formation of the blastocyst. The molecular basis of human preimplantation development is not well known, due to the scarce availability of oocytes and embryos for research. Most available knowledge is based on mouse [1], [2] or bovine [3], [4], and limited data comes from non-human primates (http://www.preger.org/). During the preimplantation phase of mammalian development, cells undergo dramatic changes. Although recent technology advances have made it possible to explore the global gene expression profiles from limited amount of material, no one has systematically explored such changes in humans. Transcription profiles of only small numbers of oocytes and embryos have been reported [5]–[8], reflecting largely the genetic profiles of individual oocytes and embryos.

In this study we thoroughly dissected more generalizable transcription profiles of large numbers of pooled morphologically normal human oocytes and embryos at six different developmental stages.

## Results and Discussion

### Overview of the transcriptome during preimplantation development: two main transitions

**Figure 1:**
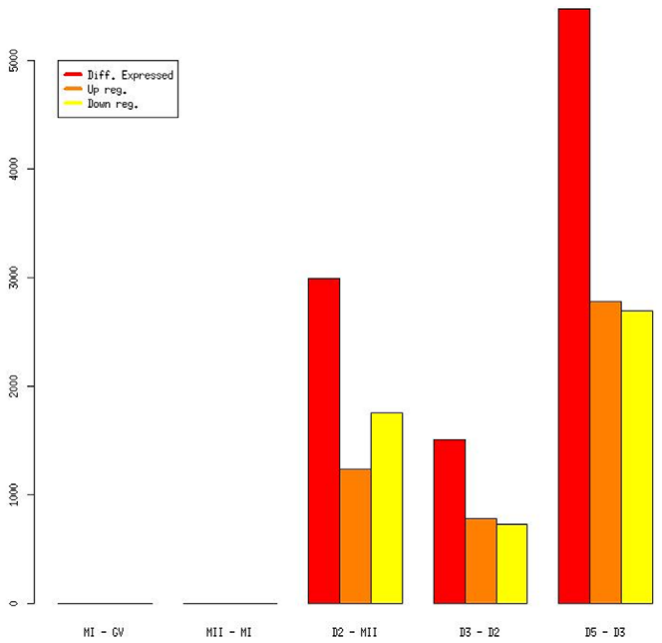
Differentially expressed probe sets between consecutive developmental stages.

The majority of the probe sets did not show statistically significant change in gene expression between developmental stages. Only 15% probe sets were up- or down regulated between stages (p<0.05). Over 80% of the differentially expressed probe sets fell into two transitions: from MII to D2 and from D3 to D5. Based on the number of the probe sets, the largest transition occurred between D3 to D5 (5477 probe sets), and the second largest between MII and D2 (2989 probe sets). 1508 probe sets were differentially expressed between D2–D3. There were no significant expression differences between the developmental stages of oocytes (GV, MI, MII) (**Fig.** 1). Using information from all developmental stages, we could cluster time series of expression levels (or sequential expression patterns) into 26 patterns (Tables S1, S16).

In the D3–D5 transition, a group of 2299 probe sets (pattern 2) were more highly expressed in D5. Gene ontology (GO, Table S18) analysis showed that these genes are significantly involved in lipid metabolic process (p-value = 10e−9), acid metabolic process (p-value = 10e−5), and fatty acid metabolic process (p-value = 10e−4) (Table S2). The higher expression of lipid metabolism in blastocysts has also been found in mice [9]. It may be related to increased cell proliferation in blastocysts, where more membrane is needed for newly forming cells. Further, the genes that had lower expression in D5 (pattern 3, 1715 probe sets) were more likely involved in transcription (p = 10e−9), regulation of nucleic acid metabolism (p = 10e−9), regulation of transcription (p = 10e−8), and spermatogenesis (p = 10e−6) (Table S3). Several well-known maternal genes were found in this set: *GDF9*, *BMP6*, *ZP1, ZP2, ZP4, POMZP3, ZAR1, NLRP5* (also known as *MATER*) and *FIGLA*. In mice, zona pellucida transcripts decrease dramatically already in D2 [1]. In our study, significant decreases of the expression of these genes were observed in D5. ZAR1 and NALP5 have been found necessary for early embryonic development in mice [10], [11]. Although their expression decreased earlier in mice than what we observed regarding the corresponding genes in human, their function in early human development may be similar.

The transition from MII to D2 consisted of 1164 probe sets with decreasing expression (pattern 4) and 691 probe sets with increasing expression (pattern 5) (Table S4 and S5). The functional assignment of the genes with decreasing expression by GO clustered them in the processes of localization (p-value = 10e−4) (Table S4, S 18). More than 70% of these transcripts were not expressed in morphologically normal human sperm (unpublished data), suggesting that they were more likely to be maternal transcripts. The proteins produced by the more highly expressed genes in the MII-D2 transition were mainly localized in nucleus (p-value = 10e−12) and ribosomes (p-value = 10e−6). GO analysis suggested that they participated in RNA processing (p-value = 10e−7), mRNA metabolism (p-value = 10e−7), and RNA splicing (p-value = 10e−8), possibly by binding RNA, or through their helicase activity (Table S5, S18). The transcriptome dynamics during MII-D2 transition fits well with the biological transition from maternal genome to zygote genome, so called MZT. MZT has been earlier, using more sporadic samples, estimated to occur around D2 and D3 in humans, and it is characterized by the activation of zygote genome and the degradation of maternal mRNA. Hence, it is comprehensible that RNA processing was highly expressed during MZT. Corresponding analyses for the enrichment in cellular compartments at the different transitions are presented in Tables S6, S7, S8, S9.

As a conclusion from all the analyzed patterns, we noticed a dramatic re-programming of transcription and translation during preimplantation development in a stage-specific manner. In the D2–D3 and D3–D5 transition, the number of transcripts that had increasing or decreasing expression was approximately the same. However, in the MII-D2 transition, more transcripts had decreasing expression than increasing expression (Table S1). This “unbalance” may due to the large scale degradation of maternal transcripts and lower number of newly activated transcripts during this stage, as also found in mice [1].

### Difference in the transcriptome between oocytes/embryos and adult tissue: autonomous preimplantation development

We also looked at the special character of the transcriptome in human oocytes and embryos by comparing our data with the profiles of human healthy adult tissue downloaded from a public database (http://www.ebi.ac.uk/arrayexpress/, E-AFMX-11). The database is generated from five tissue types: brain, kidney, heart, testis and liver (6 biological replicates each) using hgU133plus2 arrays. All the 30 tissues arrays were pooled together to represent an average adult expression level. 9,910 probe sets were expressed at a higher level in oocyte/embryo than in adult tissue, while 23,134 were expressed in at a lower level in oocyte/embryo than in adult tissue (Table S10).

**Figure 2:**
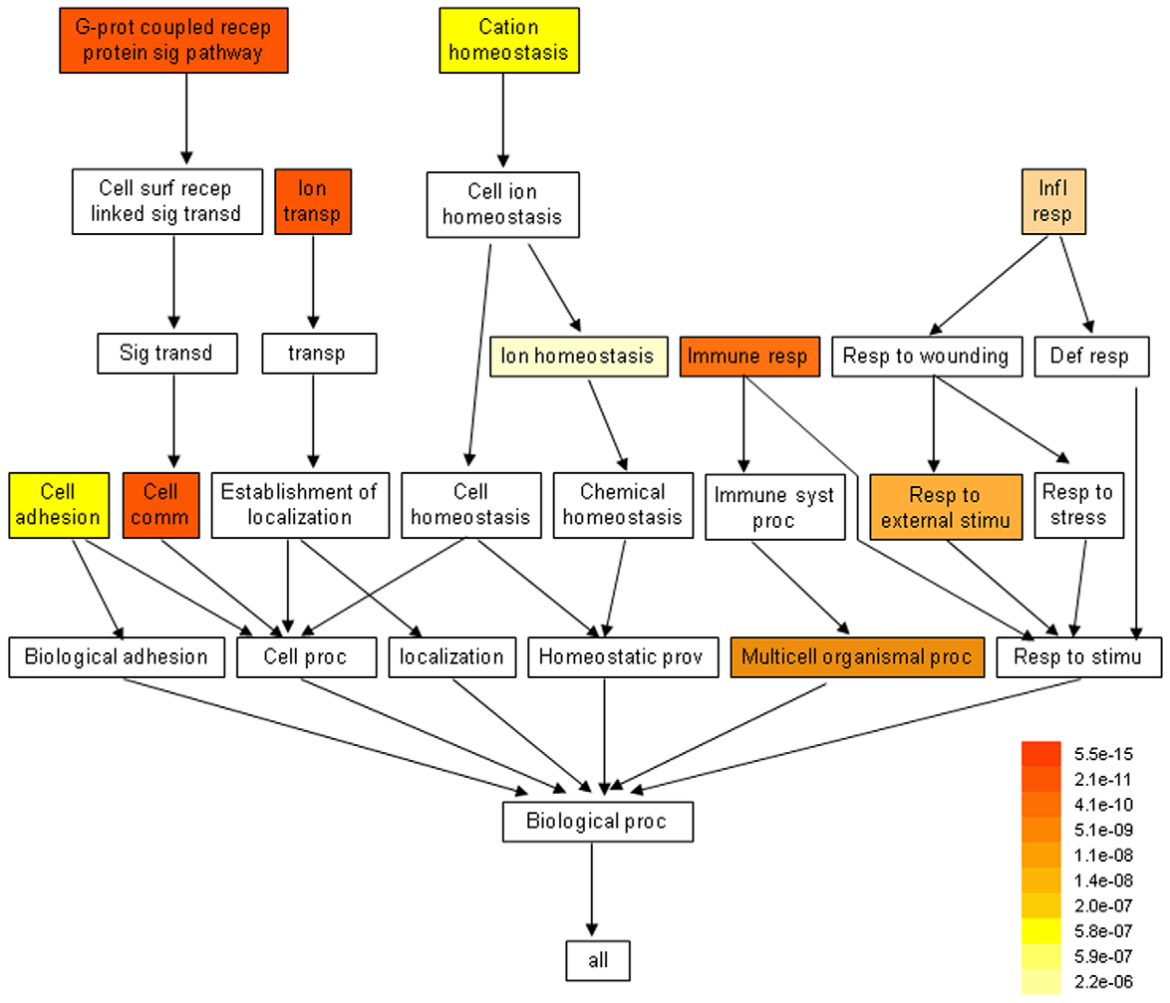
Biological Process Gene Ontology (GO) terms underrepresented in oocytes and embryos when compared with adult tissues. Colors indicate the significance (p-value) of the underrepresentation.

The more adult-specific probe sets were enriched for GO processes regulating signaling and cell communication (**Fig.** 2, Table S11): G-protein coupled receptor protein signaling pathway (p-value = e−15), cell communication (p-value = e−11), immune response (p-value = e−9), response to external stimulus (p-value = e−8), cell adhesion (p-value = e−7), sensory perception (p-value = e−4), cell surface receptor linked signal transduction (p-value = e−4). The significant underrepresentation of transcripts responsible for cell signaling and communication in oocytes and embryos indicated that human preimplantation development is almost self-directed. Hence, oocytes and early embryos proved to be self-sufficient for developmental programming before implantation because they apparently need not communicate with “outside world”, not at least with similar signaling mechanisms as the cells in adult tissues do. Our proposal supports the “quiet embryo hypothesis” indicated by Leese's group [12] who found that human preimplantation embryos have a relatively low level of metabolism. The implanted embryos take in significantly less pyruvate than those failed to implant [13]–[15]. In addition, the success of *in vitro* fertilization (IVF) in humans also supports our observation of autonomous development [16], [17]. The medium used for IVF is relatively simple and it only supplies the basic needs for cell metabolism without other special factors encountered *in vivo*. However, the oocytes and embryos are competent of development as *in vivo* and lead to healthy newborns worldwide.

In contrast to the adult-specific transcripts, the oocyte/embryo-specific transcripts were enriched for the GO terms biopolymer metabolism process (p-value = e−76), transcription (p-value = e−39), RNA biosynthetic process (p-value = e−36), nucleobase, nucleoside, nucleotide and nucleic acid metabolic process (p-value = e−33), regulation of cellular process (p-value = e−24) (Table S12). If we narrow down this “oocyte/embryo specific” list by looking at those that were more than five times higher expressed in oocyte and/or embryo, the transcripts were mainly clustered in cell division, such as mitotic cell cycle (p-value = e−12), M phase (p-value = e−11), mitosis (p-value = e−11), cell division (p-value = e−9), interphase (p-value = e−3), and spindle organization and biogenesis (p-value = e−3). These results are consistent with the notion that cell proliferation is more active during embryonic development than in adult tissues. The corresponding cellular component enrichment analyses are shown in Tables S13–S14.

### Evolution signatures of the predominated genes in oocytes/embryos: the “preimplantation genes” are generally well conserved

**Figure 3:**
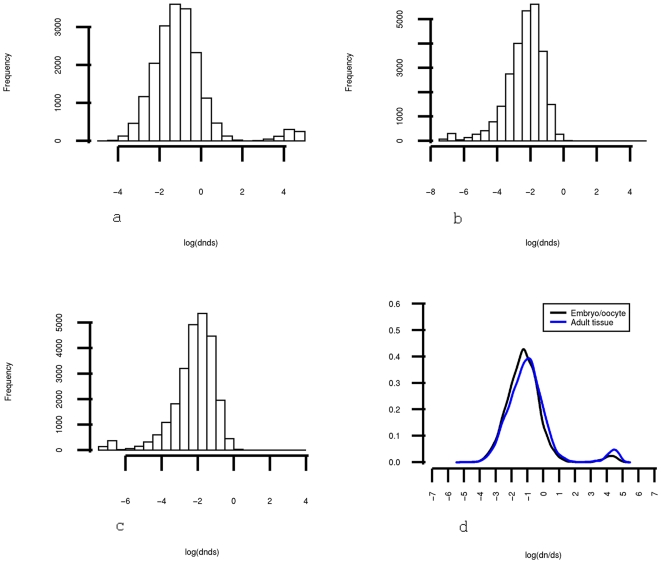
Dn/ds distribution for chimp (a), dog (b) and mice (c) for the probe sets interrogated by the HG-U133plus2 arrays. Positive selection signature can be observed in the chimp on the right side of the histogram. (d) Comparison of dn/ds for the chimp between embryo/oocyte specific and adult tissue specific probes. Testing the difference between the distributions with a nonparametric (Mann-Whitney) test yields a median shift of about 15% with a two-tailed significance p-value 10e−16.

We further characterized the genes that were more highly expressed in human oocytes and embryos when compared with adult tissues, by analyzing the evolution signatures of these genes. We used the Biomart database to access probe specific non-synonymous (dn) to and synonymous (ds) substitution rates between humans, chimps, mice and dogs. The ratio dn/ds measures the selection pressure over coding mutations: a dn/ds = 1, dn/ds<1 and dn/ds>1 imply neutral, negative and positive selection, respectively. Probes that were more highly expressed in oocytes and embryos showed on the average a dn/ds approximately 15% smaller than probes being more highly expressed in the adult tissue in all the three species (p-values between 10e−20 and 10e−30) (**Fig. 3d**), indicating a stronger selection pressure against coding mutations for these genes. The good conservation of these “preimplantation genes” obviously contributes to the continuous generation of new individuals in the four species. It has been shown that genes involved in gametogenesis tend to be under positive selection [18], [19]. Our results suggest that, not only gametogensis genes, but most genes predominantly expressed during preimplantation development are well conserved. These “preimplantation genes” are of particular interest in the field of reproductive evolution.

### Specific interesting gene: transcription factor Nr2f2

In order to highlight interesting transcription factors that may be active in the embryo development, we made a correspondence analysis between probe set expression and the motifs at the binding sites of the promoter of the genes they interrogate [20]. The transcription factor Nr2f2 was found between 3- and 5-fold more highly expressed at D5. Among the probe sets differentially regulated at D5, there was a significant overrepresentation of those harboring the binding site for Nr2f2 (p-val <10^−4^). Nr2f2 has been recently shown to mediate progesterone regulation of uterine implantation [21]. The Nr2f2-null mutant mice die during the early embryonic development due to defects in angiogenesis and heart development [22]. Heterozygote (Nr2f2 +/−) females show significantly reduced fecundity, irregular estrus cycles, delayed puberty, and retarded postnatal growth, possibly because of reduced production of progesterone and impaired uterine endometrial functions. Homozygous adult female mutants with specific inactivation of the Nr2f2 in uterine have severely impaired placental formation, leading to miscarriage at days 10–12 of pregnancy [23].

### Comparison of the transcriptome between D5 embryos and stem cell: developmental process related genes are more highly expressed in D5 embryos

Finally, to further characterize the expression profile of D5, we compared our arrays with embryonic stem cell (ESC) lines previously arrayed on HG-U133plus2 [24]. Large overrepresentation of genes annotated to developmental process (p-value = e−4), multicellular organismal processes (p-value = e−4), system development (p-value = e−3), blood vessel development (p-value = e−3), organ morphogenesis (p-value = e−3) and brain development (p-value = e−3) was found among genes that were expressed at higher level in D5 embryos than in embryonic stem cells (Table S15, S16, S17). This suggested that genes regulating implantation, placenta formation and further embryo development were active already at the blastocyst stage. Although stem cells are generated from inner cell mess of blastocyst, it seemed that the genes responsible for further embryo development had lower expression in stem cells. This could be explained by the routine supplementary of differentiation inhibitors in the culture medium.

The only ethically acceptable manner to obtain large enough numbers of human oocytes for this type of a study was to use the GV and MI oocytes which cannot be injected with sperm, and mature them in vitro. It may be that some oocytes were abnormal. They may also just be from a cohort which is at a later developmental stage by the time of initiation of the gonadotrophin stimulation. The fact that we can mature them in vitro within 24 h favours this latter possibility. In some clinical situations MII oocytes are not available, and we can utilize such in vitro matured oocytes for treatment, resulting in the birth of healthy infants. There may be some differences in the gene expression of in vivo and in vitro matured oocytes bur that difference may be small as predicted from the overall small changes during oocyte maturation. A few embryos that had not been used for clinical treatment may also have been somehow abnormal, but we did not use developmentally retarded embryos. The embryos which were frozen after the initial transfer and then not needed in treatment, were actually all of very good quality. It would have been optimal to use only good quality embryos, but that was not feasible or ethically acceptable. Minor deviations in this material may be due to the nature of our starting material, but systematic biases are unlikely as individual embryos were unlikely to have consequently similar deviations.

Some bias in the result might follow from the potentially different lengths of poly-A tails in the oocyte RNA and newly transcribed embryo RNA and subsequent difference in the efficiency of poly-T priming and reverse transcription. Such a major bias is however unlikely considering the observation that roughly similar numbers of transcripts were recorded at different stages of development, suggesting that a broad set of transcripts present at all stages were primed.

In summary, we show new original data obtained by genome wide analysis of *in vitro* matured human oocytes and embryos, revealing the almost autonomous maturation of human oocytes and early embryogenesis. We could also confirm many earlier findings based on smaller numbers of samples. Our finding and database provide a fundamental resource for the better understanding of the complex genetic network that controls early human development.

## Materials and Methods

We have had an exceptional opportunity to penetrate into the earliest events in human life by collecting, as donations for research, both large numbers of immature oocytes and preimplantation human embryos which were not used in the infertility treatment of the couples. We had ethical approval for this study from the ethics committees of Karolinska Institutet and Örebro University, Sweden. All the donating couples, who were not reimbursed, gave their informed written consent for the donation of the immature oocytes and supernumerary embryos (Supporting file S1: Materials and Methods, Figure S1, S2, S3, S4, S5, S6, S7).

**Figure 4:**
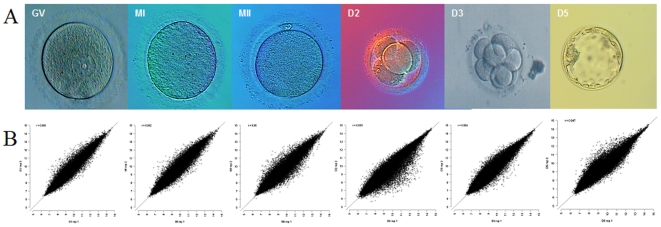
Morphology of human oocytes and embryos used in the study. B. Correlations between biological duplicates.

A total of 203 *in vitro* matured oocytes and 194 embryos were used. The six developmental stages (**Fig.** 4A) include fully-grown germinal vesicle oocyte (GV), metaphase I oocyte (MI), metaphase II oocyte (MII), 4-cell embryo (D2), 8-cell embryo (D3), and blastocyst (D5). The MII oocytes had been matured *in vitro* after donation at GV stage. D2, D3, D5 embryos were all matured *in vitro*. For each stage, we pooled 26–43 oocytes or embryos into one biological sample for RNA extraction and expression profiling (Fig. 4, Supporting file S1, Figure S1–S3). Two independent biological samples for each stage were used as replicates. Complementary DNA was amplified, and labeled according to the Affymetrix two-cycle GeneChip® Eukaryotic small sample target labeling assay (version II). The Affymetrix chip HG-U133 Plus 2.0 was used for hybridization. It was not technically feasible to make triplicates form this sparsely available material.

Data quality was assessed according to Affymetrix guidelines and benchmarks using software from the Bioconductor bundle (www.bioconductor.org). (**Fig.** 4B, Supporting online material: Materials and Methods, Figure S1, S2). To control experimental variation, the invariant set normalization method was used and expression values were extracted from PM-values using the Li-Wong method [25], in an implementation of the dChip software in R (Figure S3) [26]. Analysis of differential expression between consecutive developmental stages was performed using a Bayesian approach [27], [28] as implemented in the Limma package (www.bioconductor.org). To further characterize the pre-implantation stages, we compared those to human adult tissues hybridized still on HG-U133plus2 arrays. To account for the technical difference between adult human tissues and oocytes/embryos, the respective arrays were normalized independently, rescaled to the same median intensity and the Li-Wong method was applied to all the normalized arrays together to get summary expression measurements (http://biosun1.harvard.edu/complab/dchip/). Differential expression P-values reported were corrected for multiple testing using the FDR method.

## Supporting Information

Supporting File S1 (0.05 MB DOC)

Table S1 Regulation patterns for the oocyte and embryo maturation. The numbers +1 and −1 indicate either upregulation or downregulation between consecutive stages
(0.02 MB XLS)

Table S2 Biological Process enrichment analysis for regulation pattern 2. P-value is the significance of the enrichment, Bonf is the P-value corrected for Bonferroni. Size is the number of probesets, count the number of probesets annotated for the relative GO term and ExpCount the number expected
(0.02 MB XLS)

Table S3 Biological Process enrichment analysis for regulation pattern 3. P-value is the significance of the enrichment, Bonf is the P-value corrected for Bonferroni. Size is the number of probesets, count the number of probesets annotated for the relative GO term and ExpCount the number expected
(0.02 MB XLS)

Table S4 Biological Process enrichment analysis for regulation pattern 3. P-value is the significance of the enrichment, Bonf is the P-value corrected for Bonferroni. Size is the number of probesets, count the number of probesets annotated for the relative GO term and ExpCount the number expected
(0.02 MB XLS)

Table S5 Biological Process enrichment analysis for regulation pattern 5. P-value is the significance ofthe enrichment, Bonf is the P-value corrected for Bonferroni. Size is the number of probesets, count the number of probesets annotated for the relative GO term and ExpCount the number expected
(0.02 MB XLS)

Table S6 Cellular Component enrichment analysis for regulation pattern 2. P-value is the significance of the enrichment, Bonf is the P-value corrected for Bonferroni. Size is the number of probesets, count the number of probesets annotated for the relative GO term and ExpCount the number expected
(0.02 MB XLS)

Table S7 Cellular Component enrichment analysis for regulation pattern 3. P-value is the significance of the enrichment, Bonf is the P-value corrected for Bonferroni. Size is the number of probesets, count the number of probesets annotated for the relative GO term and ExpCount the number expected
(0.01 MB XLS)

Table S8 Cellular Component enrichment analysis for regulation pattern 4. P-value is the significance of the enrichment, Bonf is the P-value corrected for Bonferroni. Size is the number of probesets, count the number of probesets annotated for the relative GO term and ExpCount the number expected
(0.01 MB XLS)

Table S9 Cellular Component enrichment analysis for regulation pattern 5. P-value is the significance of the enrichment, Bonf is the P-value corrected for Bonferroni. Size is the number of probesets, count the number of probesets annotated for the relative GO term and ExpCount the number expected
(0.02 MB XLS)

Table S10 Comparison of oocytes/embryo with pooled adult tissues. The numbers +1 and −1 indicate either upregulation or downregulation between the samples
(0.01 MB XLS)

Table S11 Biological Process enrichment analysis for oocytes/embryo to adult tissues comparison, pattern 1. P-value is the significance of the enrichment, Bonf is the P-value corrected for Bonferroni. Size is the number of probesets, count the number of probesets annotated for the relative GO term and ExpCount the number expected
(0.03 MB XLS)

Table S12 Biological Process enrichment analysis for oocytes/embryo to adult tissues comparison, pattern 3. P-value is the significance of the enrichment, Bonf is the P-value corrected for Bonferroni. Size is the number of probesets, count the number of probesets annotated for the relative GO term and ExpCount the number expected
(0.03 MB XLS)

Table S13 Cellular Component enrichment analysis for oocytes/embryo to adult tissues comparison, pattern 1. P-value is the significance of the enrichment, Bonf is the P-value corrected for Bonferroni. Size is the number of probesets, count the number of probesets annotated for the relative GO term and ExpCount the number expected
(0.02 MB XLS)

Table S14 Cellular Component enrichment analysis for oocytes/embryo to adult tissues comparison, pattern 3. P-value is the significance of the enrichment, Bonf is the P-value corrected for Bonferroni. Size is the number of probesets, count the number of probesets annotated for the relative GO term and ExpCount the number expected
(0.01 MB XLS)

Table S15 Biological Process enrichment analysis for oocytes/embryo to stem cells comparison. P-value is the significance of the enrichment, Bonf is the P-value corrected for Bonferroni. Size is the number of probesets, count the number of probesets annotated for the relative GO term and ExpCount the number expected
(0.01 MB XLS)

Table S16 Affymetrix transcripts at different stages
(6.21 MB XLS)

Table S17 Affymetrix transcripts at different stages, part II
(4.89 MB XLS)

Table S18 Gene ontology cathegories
(2.73 MB ZIP)

Figure S1 Figure S1 QC, Spikes-In and RNA degradation plots
(9.93 MB TIF)

Figure S2 Intensities for raw data (a) and (b) and for the normalized data (c)
(9.93 MB TIF)

Figure S3 Correlation between chips (right) and hierarchical clustering of the arrays (left) based on the Pearson correlation coefficient
(8.87 MB TIF)

Figure S4 Correlation between replicates. The expression values of the repilcates (in log2 scale) are plotted against each other
(1.40 MB TIF)

Figure S5 High and low expressed probe sets for p-value = 0.05 (left), p-value = 0.005 (center) and p-value = 0.0005 (right). Yellow bars represent the number of probe sets with lower expression, the orange those with higher expression and the red ones the sum of the two
(9.93 MB TIF)

Figure S6 High and low expressed probe sets for p-value = 0.05 (left), p-value = 0.005 (center) and p-value = 0.0005 (right). Yellow bars represent the number of probe sets with lower expression, the orange those with higher expression and the red ones the sum of the two.
(9.93 MB TIF)

Figure S7 Differential expression in mice. Yellow bars represent the number of probe sets with lower expression, the orange those with higher expression and the red ones the sum of the two.
(6.75 MB TIF)

## References

[1] Wang QT, Piotrowska K, Ciemerych MA, Milenkovic L, Scott MP (2004). A genome-wide study of gene activity reveals developmental signaling pathways in the preimplantation mouse embryo.. Dev Cell.

[2] Hamatani T, Carter MG, Sharov AA, Ko MS (2004). Dynamics of global gene expression changes during mouse preimplantation development.. Dev Cell.

[3] Ushizawa K, Herath CB, Kaneyama K, Shiojima S, Hirasawa A (2004). cDNA microarray analysis of bovine embryo gene expression profiles during the pre-implantation period.. Reprod Biol Endocrinol.

[4] Mamo S, Sargent CA, Affara NA, Tesfaye D, El-Halawany N (2006). Transcript profiles of some developmentally important genes detected in bovine oocytes and in vitro-produced blastocysts using RNA amplification and cDNA microarrays.. Reprod Domest Anim.

[5] Kocabas AM, Crosby J, Ross PJ, Otu HH, Beyhan Z (2006). The transcriptome of human oocytes.. Proc Natl Acad Sci U S A.

[6] Zhang P, Kerkela E, Skottman H, Levkov L, Kivinen K (2006). Distinct sets of developmentally regulated genes that are expressed by human oocytes and human embryonic stem cells.. Fertil Steril.

[7] Dobson AT, Raja R, Abeyta MJ, Taylor T, Shen S (2004). The unique transcriptome through day 3 of human preimplantation development.. Hum Mol Genet.

[8] Assou S, Anahory T, Pantesco V, Le Carrour T, Pellestor F (2006). The human cumulus–oocyte complex gene-expression profile.. Hum Reprod.

[9] Cui XS, Li XY, Shen XH, Bae YJ, Kang JJ (2006). Transcription profile in mouse four-cell, morula, and blastocyst: Genes implicated in compaction and blastocoel formation.. Mol Reprod Dev.

[10] Wu X, Viveiros MM, Eppig JJ, Bai Y, Fitzpatrick SL (2003). Zygote arrest 1 (Zar1) is a novel maternal-effect gene critical for the oocyte-to-embryo transition.. Nat Genet.

[11] Tong ZB, Gold L, Pfeifer KE, Dorward H, Lee E (2000). Mater, a maternal effect gene required for early embryonic development in mice.. Nat Genet.

[12] Leese HJ (2002). Quiet please, do not disturb: a hypothesis of embryo metabolism and viability.. Bioessays.

[13] Houghton FD, Hawkhead JA, Humpherson PG, Hogg JE, Balen AH (2002). Non-invasive amino acid turnover predicts human embryo developmental capacity.. Hum Reprod.

[14] Turner K, Martin KL, Woodward BJ, Lenton EA, Leese HJ (1994). Comparison of pyruvate uptake by embryos derived from conception and non-conception natural cycles.. Hum Reprod.

[15] Conaghan J, Hardy K, Handyside AH, Winston RM, Leese HJ (1993). Selection criteria for human embryo transfer: a comparison of pyruvate uptake and morphology.. J Assist Reprod Genet.

[16] O'Brien MJ, Pendola JK, Eppig JJ (2003). A revised protocol for in vitro development of mouse oocytes from primordial follicles dramatically improves their developmental competence.. Biol Reprod.

[17] Eppig JJ, O'Brien MJ (1996). Development in vitro of mouse oocytes from primordial follicles.. Biol Reprod.

[18] Nielsen R, Bustamante C, Clark AG, Glanowski S, Sackton TB (2005). A scan for positively selected genes in the genomes of humans and chimpanzees.. PLoS Biol.

[19] Bustamante CD, Fledel-Alon A, Williamson S, Nielsen R, Hubisz MT (2005). Natural selection on protein-coding genes in the human genome.. Nature.

[20] Jeffery IB, Madden SF, McGettigan PA, Perriere G, Culhane AC (2007). Integrating transcription factor binding site information with gene expression datasets.. Bioinformatics.

[21] Kurihara I, Lee DK, Petit FG, Jeong J, Lee K (2007). COUP-TFII mediates progesterone regulation of uterine implantation by controlling ER activity.. PLoS Genet.

[22] Pereira FA, Qiu Y, Zhou G, Tsai MJ, Tsai SY (1999). The orphan nuclear receptor COUP-TFII is required for angiogenesis and heart development.. Genes Dev.

[23] Petit FG, Jamin SP, Kurihara I, Behringer RR, DeMayo FJ (2007). Deletion of the orphan nuclear receptor COUP-TFII in uterus leads to placental deficiency.. Proc Natl Acad Sci U S A.

[24] Zhang P, Kerkela E, Skottman H, Levkov L, Kivinen K (2007). Distinct sets of developmentally regulated genes that are expressed by human oocytes and human embryonic stem cells.. Fertil Steril.

[25] Li C, Hung Wong W (2001). Model-based analysis of oligonucleotide arrays: model validation, design issues and standard error application.. Genome Biol.

[26] Li C, Wong WH (2001). Model-based analysis of oligonucleotide arrays: expression index computation and outlier detection.. Proc Natl Acad Sci U S A.

[27] Smyth GK, Smyth GK, RG, Carey V, Dudoit S, Irizarry R, Huber W (2005). Limma: linear models for microarray data.. Bioinformatics and Computational Biology Solutions using R and Bioconductor.

[28] Smyth GK (2004). Linear models and empirical bayes methods for assessing differential expression in microarray experiments.. Stat Appl Genet Mol Biol.

