# Supplementary material for: Transcriptome Profiling of Human Pre-Implantation Development
Source: PLoS One. 2009 Nov 16;4(11):e7844. doi: 10.1371/journal.pone.0007844 (PMC2773928; doi:10.1371/journal.pone.0007844)
Supplement: Supporting File S1 — (0.05 MB DOC) [file pone.0007844.s001.doc]

Supporting file S1

1 SECTION 1: MATERIALS AND METHODS

This study was approved by the Ethics Committees of Karolinska Institutet, Karolinska University Hospital Huddinge Örebro University Hospital and Uppsala University Hospital. Informed consent was obtained from all oocyte and embryo donors. None of the donors received any financial re-imbursement.

- 1. Collection and culture of human oocytes

Morphologically healthy GV and MI oocytes were donated by women at the Fertility Unit, Karolinska University Hospital Huddinge. MII oocytes were obtained by in vitro maturation of such donated GV oocytes. The age (mean ± SD) of the women was 35 ± 2 years (range 25 - 39 years). These healthy women underwent intracytoplasmic sperm injection (ICSI) treatment due to severe male factor infertility.

Only mature oocytes can be injected with sperm, and the immature ones are normally discarded. All these women had mature oocytes for ICSI, and they donated immature ones to our study. They had undergone hormone stimulation using a gonatropin releasing hormone (GnRH) agonist or antagonist, FSH and hCG before oocyte retrieval. The oocytes were denuded to be cumulus-free by hyaluronidase solution (HYASE-10X, Vitrolife, Gothenburg, Sweden), and the immature oocytes (GV and MI) were colleted for our study.

For maturing the GV oocytes, we used a similar in vitro culture system as that used in our clinic for in vitro maturation (IVM) of oocytes. The clinic IVM has led to pregnancies and healthy infants. The basic medium is Tissue Culture Medium199 (Sigma, St. Louis, MO, USA), supplemented with 10% patients serum, 0.3 mM pyruvate (Sigma; St. Louis, MO), 0.075 IU/ml FSH and 0.5 IU/ml HCG (Serono, Rome, Italy). In most cases, GV oocytes matured into MII within 24 hours. If the oocytes did not development to MII after 48 hours they were not used in this study. Oocytes were washed in RNase-free PBS three times before stored in 100-200 μl of RNA later (Ambion, Cambridge, UK) at -70 .C. Prior to RNA isolation, oocytes were first thawed at room temperature within RNAlater, and then removed from RNAlater to RNase-free PBS and

washed three times before moved in RNA lyses buffer (Qiagen, Hilden, Germany).

1.2 Collection and culture of human embryos

The embryos were obtained as donations from two in vitro Fertilization Units in Örebro University Hospital and Uppsala University Hospital. Only embryos that would not be used in the couples’ infertility treatment were used for this study. The donated embryos had been frozen for possible infertility treatment in the future after the couple had had the best 1-2 embryos transferred on day two or three after fertilization. When the couples did not desire to use them in treatment any more, they donated the embryos to this study. Both partners gave their informed consent for the donation after receiving oral and written information, and they did not receive any financial re-imbursement. The researchers of this study did not participate in asking for the consent. Down-regulation for ovarian hyperstimulation was achieved using a long protocol gonadotropin releasing hormone agonist (GnRHa), nafarelin (Synarela; Syntex Nordica AB, Södertälje,

Sweden) administered intra-nasally, starting on either cycle day 1 or 21. Following down-regulation, ovarian stimulation was induced using a recombinant FSH, (rFSH; Gonal-F, Serono laboratories, Aubonne, Switzerland or, rFSH; Puregon, NY Organon, Oss, The Netherlands). The starting dose was dependent on the patient’s age and/or previous response to ovarian stimulation. Ovarian response was monitored by serum estradiol assays and vaginal ultrasound scans. GnRHa and rFSH were administered until the leading follicle had a diameter of at least 18 mm. Maturation of the oocyte was triggered by one s.c. injection of 10000 IU of

human chorionic gonadotrophin, (hCG; Profasi, Serono laboratories, Aubonne, Switzerland). Thirty-seven hours after hCG administration, oocytes were retrieved by trans-vaginal needle aspiration under ultrasound guidance.

Conventional IVF was performed in 20 ml medium (IVF, Vitrolife AB, Gothenburg, Sweden)

droplets under oil containing about 15000 spermatozoa (IVF, Ovoil, Vitrolife AB, Gothenburg, Sweden). For intracytoplasmic sperm injection (ICSI), oocytes were stripped of cumulus cells by mechanical pipetting after brief exposure to hyaluronidase (HYAS; Vitrolife AB, Gothenburg, Sweden). ICSI was then performed using a Nikon-Narishige micromanipulation system. Fertilization was evaluated 18-20 hours after insemination. Following fertilization IVF and ICSI embryos were cultured in 10 ml droplets of medium under oil (G.1.2; Vitrolife AB, Gothenburg, Sweden). Embryo transfer was done either on day 2 or day 3. Excess embryos, surplus to treatment were frozen at the 2-8-cell stage using a three-stage propanediol cryopreservation

kit (Freeze kit 1; Vitrolife AB, Gothenburg, Sweden) according to the manufacturer’s instructions. Embryos used for this study were thawed (Sydney IVF thawing kit, CooK IVF, Brisbane, Australia) and used as such at cleavage stage (N = 134) or cultured to blastocyst stage (N = 60) in either BlastAssist System (Medicult, Jyllinge, Denmark) or blastocyst sequential media (Syidney IVF Blastocyst medium, CooK IVF, Brisbane, Australia). Prior to RNA isolation, embryos were first washed three times in RNase-free PBS, then transferred to 50-100 μl of RNA lyses buffer (Qiagen, Hilden, Germany) and stored at -70 .C.

**1.3 RNA isolation and oligonucleotide microarray**

After isolation using an RNeasy mini kit (Qiagen, Hilden, Germany), total RNA (50 ng for all samples) was reverse transcribed, amplified, labeled and hybridized according to the Affymetrix two-cycle GeneChip Eukaryotic small sample target labeling assay version II (Affymetrix; Santa Clara, CA, USA). 20 μg of biotin-labeled cRNA was fragmented and 15μg was hybridized to HG-U133 Plus 2.0 array (Affymetrix, INC. Santa Clara, CA, USA). HG-U133 Plus 2.0 array provides a comprehensive coverage of the transcribed human genome on a single array. It analyzes the expression level of over 47,000 transcripts and variants, including 38,500 well-characterized human genes. Arrays were stained and scanned according to Affymetrix protocols. Experiments were performed in duplicates for each stage.

2 Data Analysis

2.1 Quality control

The quality of the chips was assessed using the guidelines and the benchmarks of Affymetrix specific for the amplification procedure (Affymetrix two-cycle GeneChip Eukaryotic small sample target labeling assay II). We used the software package simpleaffy V2.8.0 from the Bioconductor repository in order to estimate background, scale factors, percentage of present probe sets and GAPDH and B-Actin expression levels ratios at 3 prime, middle position (M), and 5 prime. Background values were in the 20-100 range as expected. Percentage of present probe sets was between 36%-42%, which is consistent with Affymetrix benchmark of 40%. GAPDH and B-Actin 3prime/M ratio was below 3 for all the chips, as recommended (figure S1(a)). Linearity of the target

amplification was checked by using the Affymetrix Spike-in. No sensible deviation from linearity was reported (figure S1(b)). The BioB probe was called present on all the chips, as suggested. Finally, the quality of the RNA was checked by plotting the chip average probe intensity versus the probe position (probe number). No sensible deviation from linearity was noticed (figure S1(c)).

2.2 Normalization

Normalization of the data was done using the package affy 1.12.1 from the Bioconductor repository (www.bioconductor.org). We used only PM probes, invariant set, no background subtraction and the Li-Wong [3] summary method. This choice mimics the MBEI algorithm used in the dCHIP software (www.dchip.org). The probe intensities before and after normalization were showed in figure S 2. The study was designed with two biological replicates for each of the developmental stages. The overall similarity of the biological replicates was assessed by the Pearson correlation coefficient r. This was r > 0.93 for all the replicates pairs. The dissimilarity between consecutive stages, measured by then metric d = 1 − r^2^ (figure S 3) showed the largest changes at the transition of D2-MII and D3-D5. We also compared all the arrays together by hierarchical clustering: biological replicates come next to each other on the leaves of the resulting dendogram except for the GV oocyte and Metaphase I stages, reflecting the similarity genes between the two stages (figure S 3).

The correlations of expression levels between two replicate samples are shown in figure S 4.

3 Probe sets expression

The time profiled differential expression of genes was assessed using the hierarchical fitting method implemented in R. The software package LIMMA 2.9.1 (www.bioconductor.org) was used. The multiple testing problem was approached using the Benjamini & Hochberg [1] method to correct the single gene high/low expression p-values. We used a threshold of p-value = 0.05 (after correction) to record a gene as differentially expressed between two consecutive stages. The impact of changes in this threshold is shown in figure S 5, where the number of differentially expressed probe sets are histogrammed for p-value=0.05, 0.005 and 0.0005 in figure S 5.

3.1 Gene Ontology (GO) Enrichment Analysis

Totally, 46,464 probe sets did not show any differential expression, while 8211 showed changes between MII and Day 2, Day 2 and Day 3 or Day3 and Day 5, table S 1. We mapped the pattern specific genes to the Gene Ontology (GO) and KEGG pathway databases and looked for terms either over or under represented using the package GOSTATS 1.7.4 (www.bioconductor.org). The probe sets on the hgu133plus2 microarrays were mapped to the ENTREZ database and the gene lists obtained filtered eliminating both the probe sets that had not either annotation or mapping to GO and KEGG databases. Gene Ontology tests were based on the hyper geometric distribution and were conditioned on the structure of the GO graphs.

The universe (or reference group) was chosen to be the union of the genes that showed any differential expression (see also http://www.bioconductor.org/packages/2.0/bioc/vignettes/GOstats/inst/doc/

GOstatsHyperG.pdf). The GO assay results of pattern 2-5 are shown in the tables S 2- 9.

4 General LIMMA model to test the differential expression between

embryo and adult

A total of 30 hgU133plus2 arrays with tissues from healthy adults were downloaded from the public database (http://www.ebi.ac.uk/arrayexpress/E-AFMX-11) in order to compare them with our human embryo arrays. Tissues hybridized were brain, kidney, heart, testis and liver (6 biological replicates each). To control experimental variation, the invariant set normalization method was used and expression values were extracted from PM-values using the Li-Wong [3] method , in an implementation of the dChip software in R. Analysis of differential expression between consecutive developmental stages was performed using a Bayesian approach as implemented in the Limma package (www.bioconductor.org). Searching for differential

expression, the adult tissues arrays were pooled together to represent an average adult expression levels for the genes targeted in the Affymetrix arrays. GO assay of the high and low expressed transcripts are shown in the tables S 10-14.

5 Comparison with other species

We used the BIOMART database www.biomart.org to annotate the probes on the arrays with the dn/ds ratio, which measures natural selection at the protein level. Probe sets with no annotation were eliminated from further analysis. Species used were chimp, dog and mouse (figure S 6). We compared ds/ds values for all the probes low expressed with dn/ds values for all the probes high expressed from embryo to adults on the three specific species. We found that: for chimp, the median values difference was -0.048 (p-value=10^−16^), for the dog the median difference was -0.019 (p-value=10^−27^) and for mouse the median values difference was -0.015 (p-value=10^−27^). These findings support the hypothesis that embryo specific genes undergo a larger

selection pressure at the protein level. To further strengthen this argument, we annotated the probe sets with the number of synonymous and non synonymous variations and estimated the probe set wise ratio of these two values, nnsyn/nsyn. When comparing the embryo specific with the adult specific probe sets, we found that the ratio is about 5% smaller for probe sets high expressed in embryos when compared with those high expressed in adult tissues (p-value=0.001).

6 Comparison with data generated from mice

Microarrays for 12 different phases of preimplatation embryo development (GV, MII, zygote, early 2 cells, mid 2 cells, late 2 cells, 4 cells, 8 cells, 16 cells, early blastocyst, mid blastocyst, late blastocyst) were available from the public database (http://www.ebi.ac.uk/arrayexpress/experimentE-MEXP-51).

Arrays used are Affymetrix mgU74av2 which targets 12,488 genes. Replicating our analysis in these samples we found that much more differential expression was present in the mouse (figure S 7). The genes that showed no changes in regulation accounted for 44% of the total (85% in human embryo) while the most consistent differential expression was between germinal vesicle and metaphase II with approximately 4000 genes (no changes were observed in human embryos).

7 Comparison of Day 5 with cultured embryonic stem cells

The derivation and characterization of the hESC used is described elsewhere [4]. RNA was arrayed on HG-U133plus2 arrays. Normalization and QC proceeded as described above. QC parameters where within the thresholds suggested by Affymetrix. Expression levels of probe sets high expressed at Day 5 where compared with expression levels for the same probe sets in the hESC arrays and then enrichment analysis was performed as described above. Results are reported in table S 15.

8 Transcription Factor Binding Sites Analysis

Transcription factor binding sites (TFBS) where mined using a co-inertia method on expression values and TFBS motifs annotated to the probe sets. The analysis is based on the work of Jeffery et al.[2] and the annotations were obtained from the authors. By setting the threshold to p-value = 0.05, we got about 16 motifs that correlated with differentially expressed genes at Day 5. Out of these, 8 could be checked for differential expression, since there were probe sets interrogating them on the same arrays. Only one factor, namely NR2F2, was significantly differentially regulated in Day 5, with higher expression between 3 and 5 fold over 5 probe sets.

References

[1] Yoav Benjamini and Yosef Hochberg. Controlling the false discovery rate: A practical and powerful approach to multiple testing. Journal of the Royal Statistical Society. Series B (Methodological), 57(1):289–300, 1995.

[2] Ian B. Jeffery, Stephen F. Madden, Paul A. McGettigan, Guy Perriere, Aedin C. Culhane, and

Desmond G. Higgins. Integrating transcription factor binding site information with gene expression datasets. Bionformatics, 23:298–305, 2007.

[3] Cheng Li andWing HungWong. Model-based analysis of oligonucleotide arrays: Model validation, design issues and standard error application. Genome Biology, 2(8):RESEARCH0032, 2001.

[4] Pu Zhang, Erja Kerkela, Levi Skottman, Lev Levkov, Katja Kivinen, Riitta Lahesmaa, Outi Hovatta, and Kere Juha. Distinct sets of developmentally regulated genes that are expressed by human oocytes and human embryonic stem cells. Fertility and Sterility, 87:677–690, 2007.
