# Supplementary figures and images for: Transcriptome Profiling of Human Pre-Implantation Development
Source: PLoS One. 2009 Nov 16;4(11):e7844. doi: 10.1371/journal.pone.0007844 (PMC2773928; doi:10.1371/journal.pone.0007844)

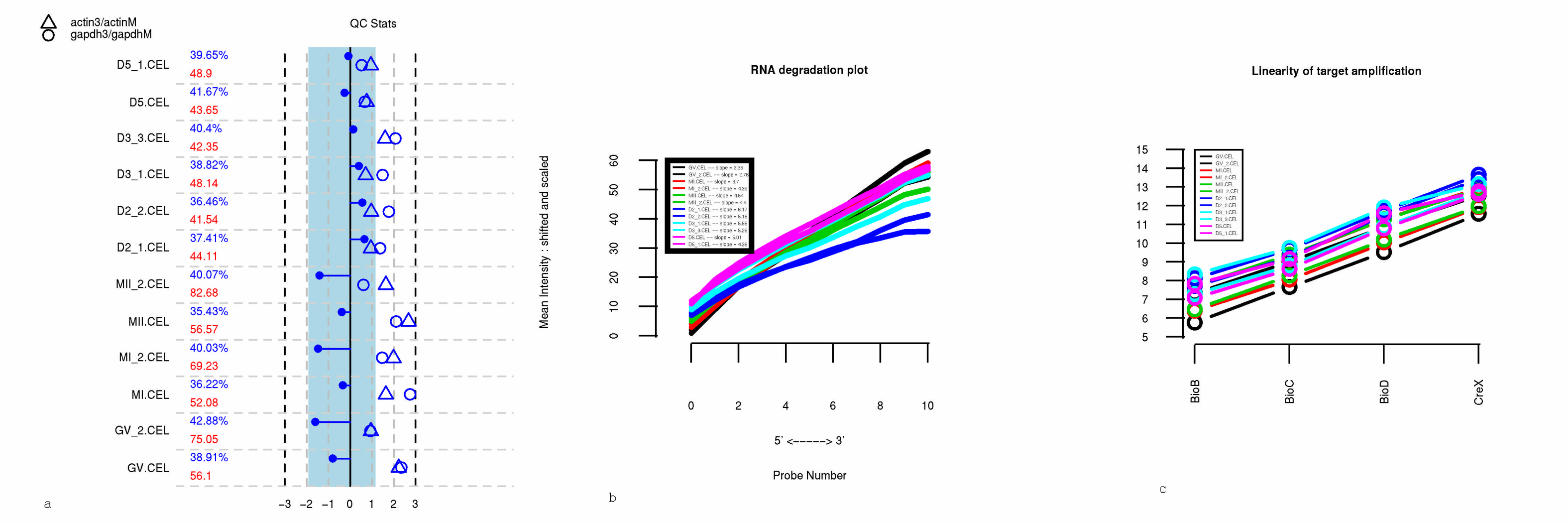

Supplement: Figure S1 — QC, Spikes-In and RNA degradation plots (9.93 MB TIF) [file pone.0007844.s020.tif]

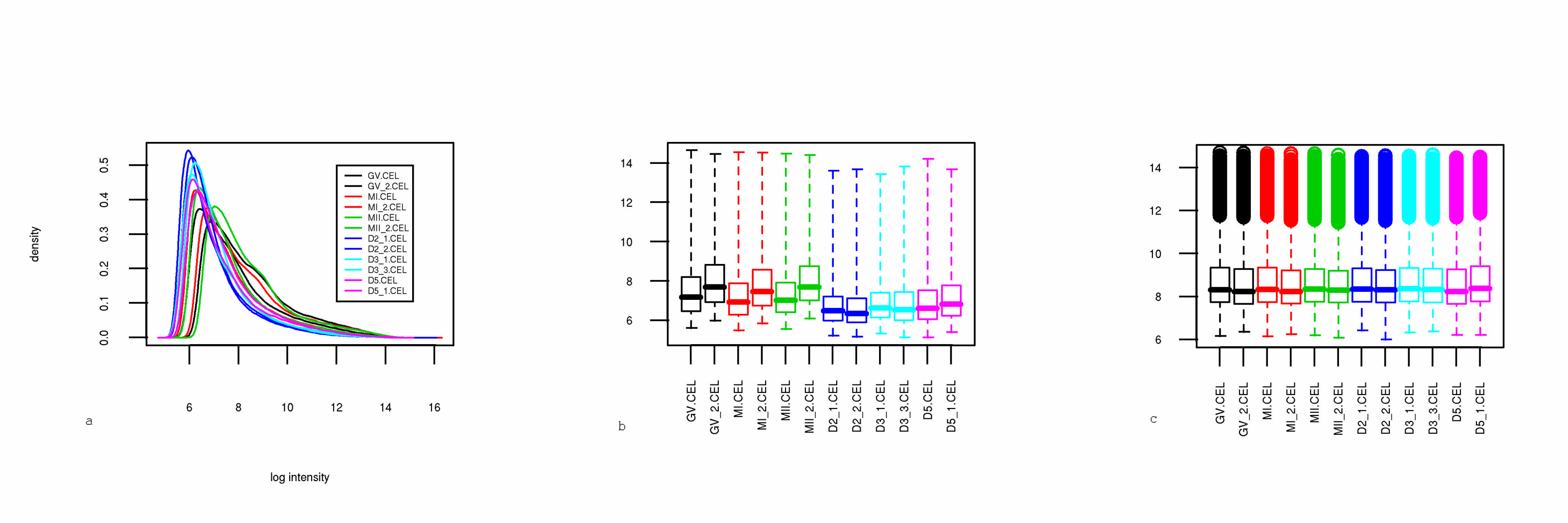

Supplement: Figure S2 — Intensities for raw data (a) and (b) and for the normalized data (c) (9.93 MB TIF) [file pone.0007844.s021.tif]

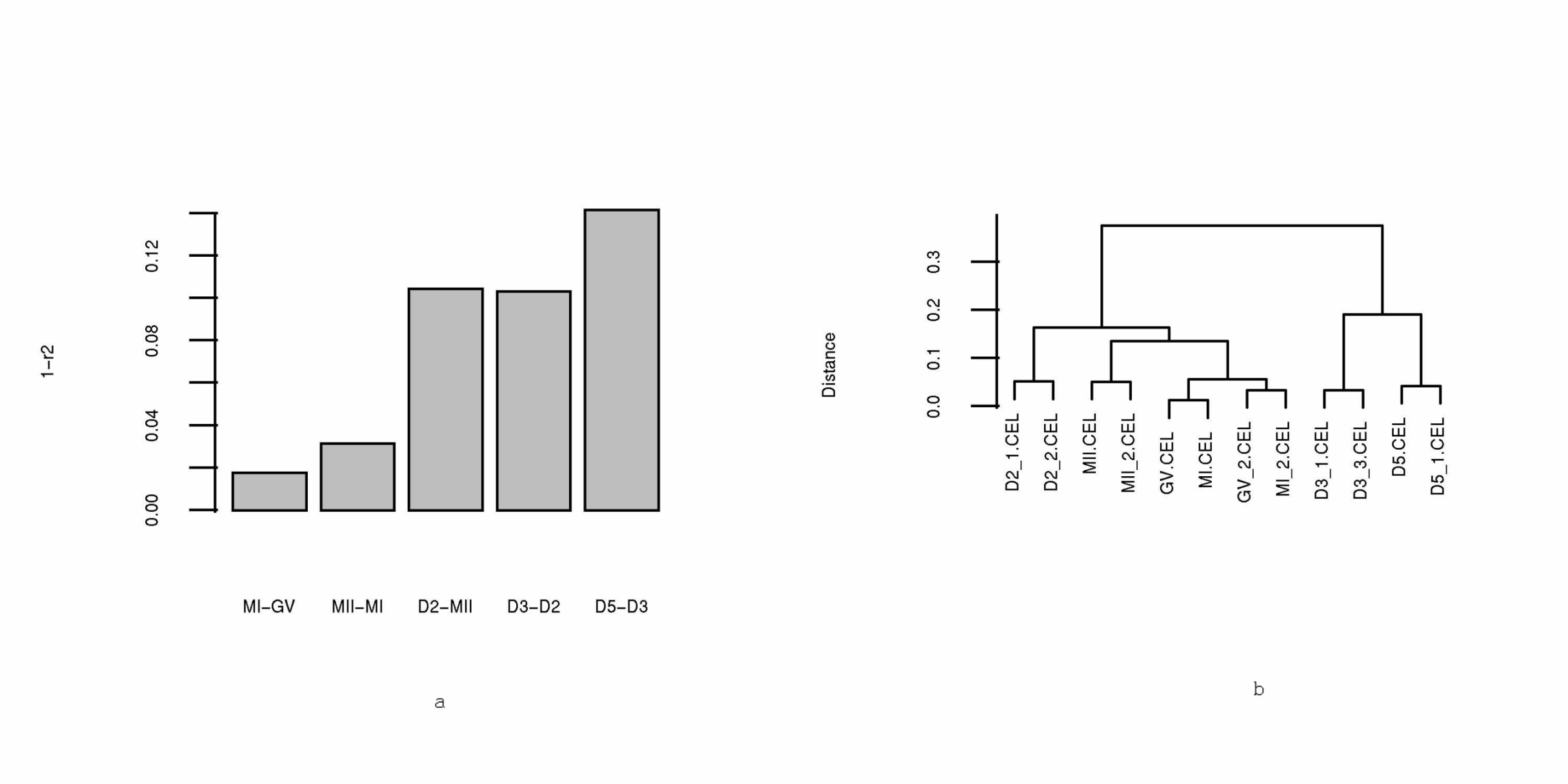

Supplement: Figure S3 — Correlation between chips (right) and hierarchical clustering of the arrays (left) based on the Pearson correlation coefficient (8.87 MB TIF) [file pone.0007844.s022.tif]

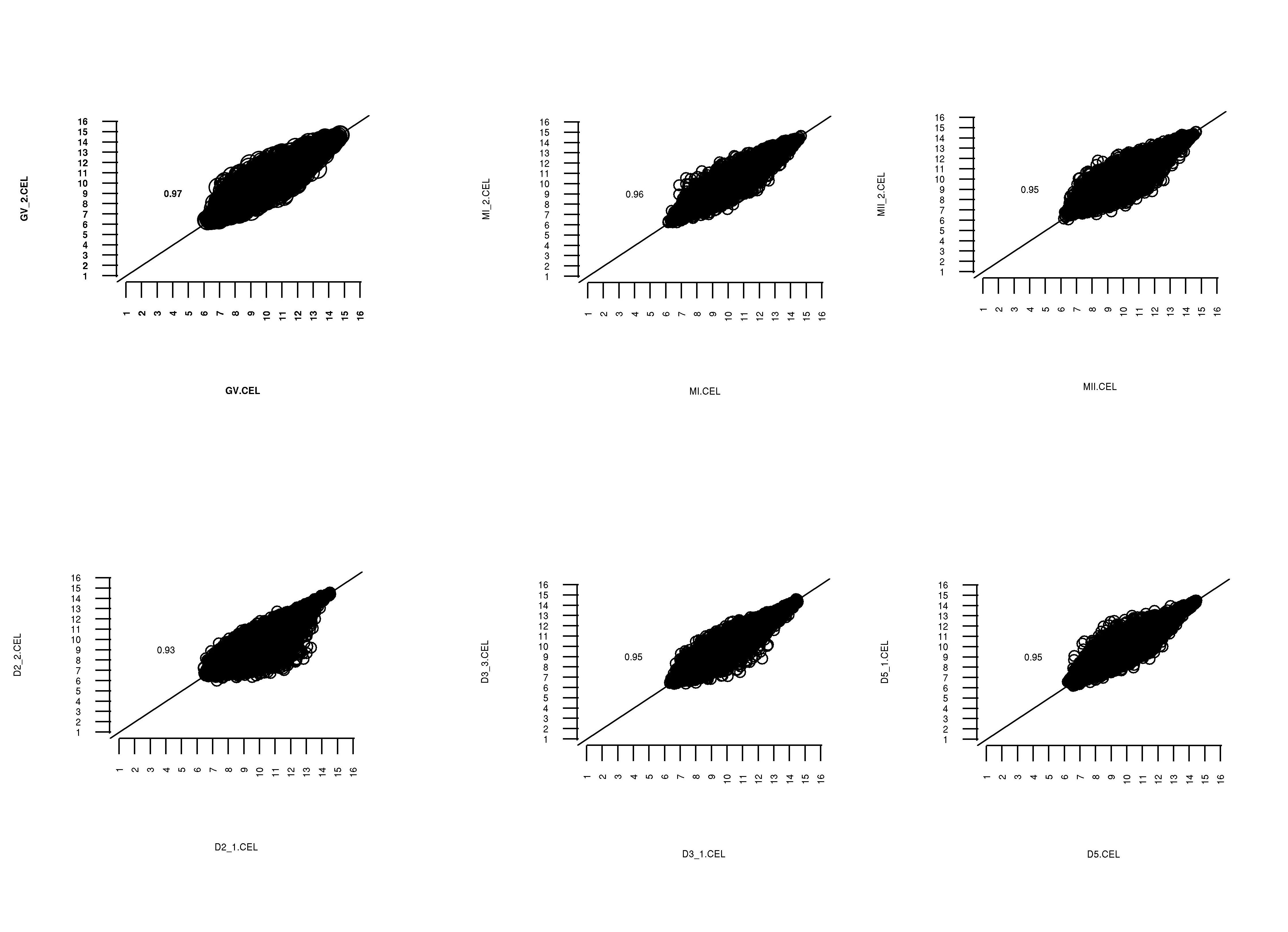

Supplement: Figure S4 — Correlation between replicates. The expression values of the repilcates (in log2 scale) are plotted against each other (1.40 MB TIF) [file pone.0007844.s023.tif]

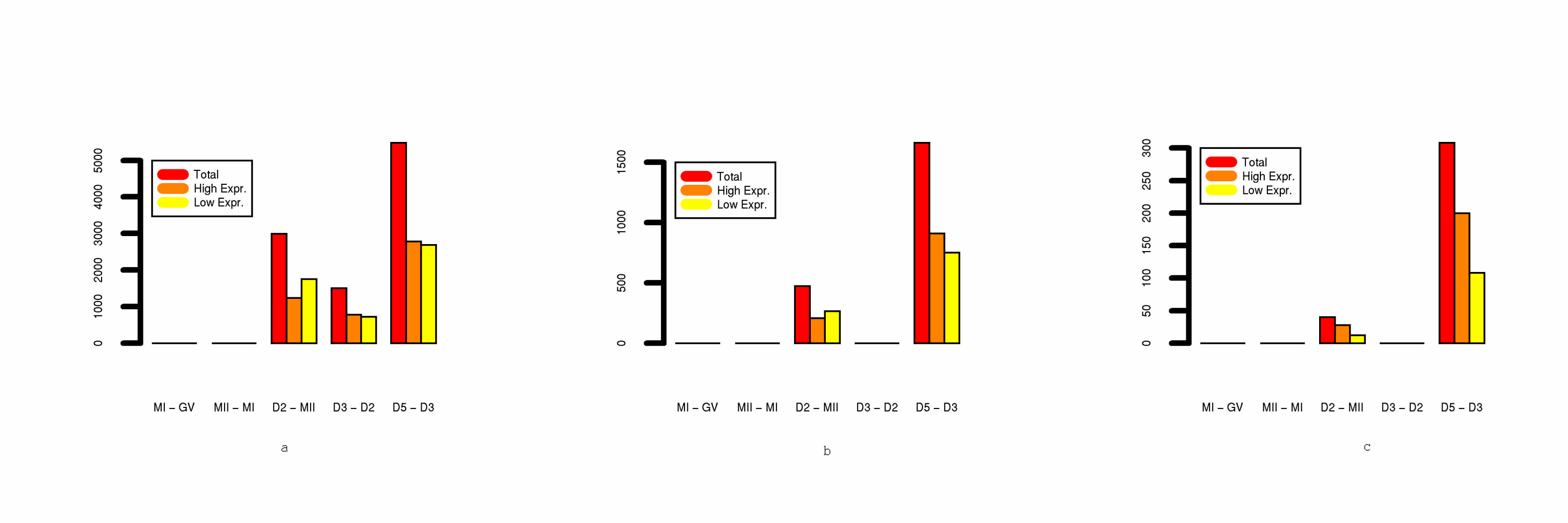

Supplement: Figure S5 — High and low expressed probe sets for p-value = 0.05 (left), p-value = 0.005 (center) and p-value = 0.0005 (right). Yellow bars represent the number of probe sets with lower expression, the orange those with higher expression and the red ones the sum of the two (9.93 MB TIF) [file pone.0007844.s024.tif]

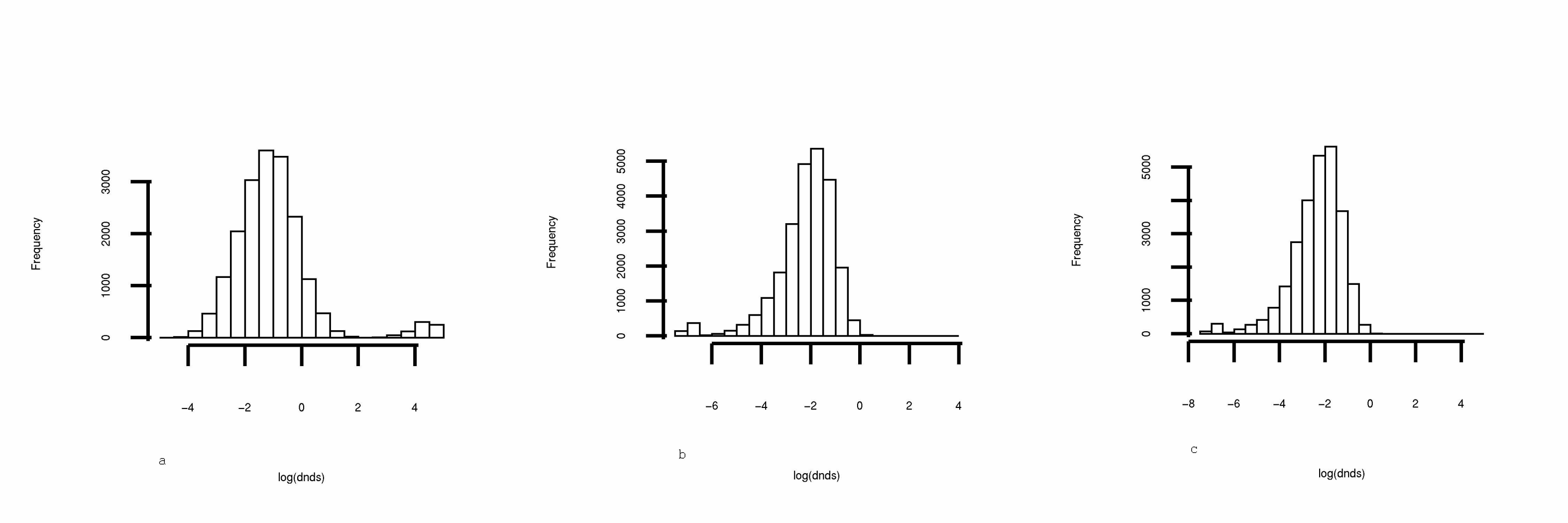

Supplement: Figure S6 — High and low expressed probe sets for p-value = 0.05 (left), p-value = 0.005 (center) and p-value = 0.0005 (right). Yellow bars represent the number of probe sets with lower expression, the orange those with higher expression and the red ones the sum of the two. (9.93 MB TIF) [file pone.0007844.s025.tif]

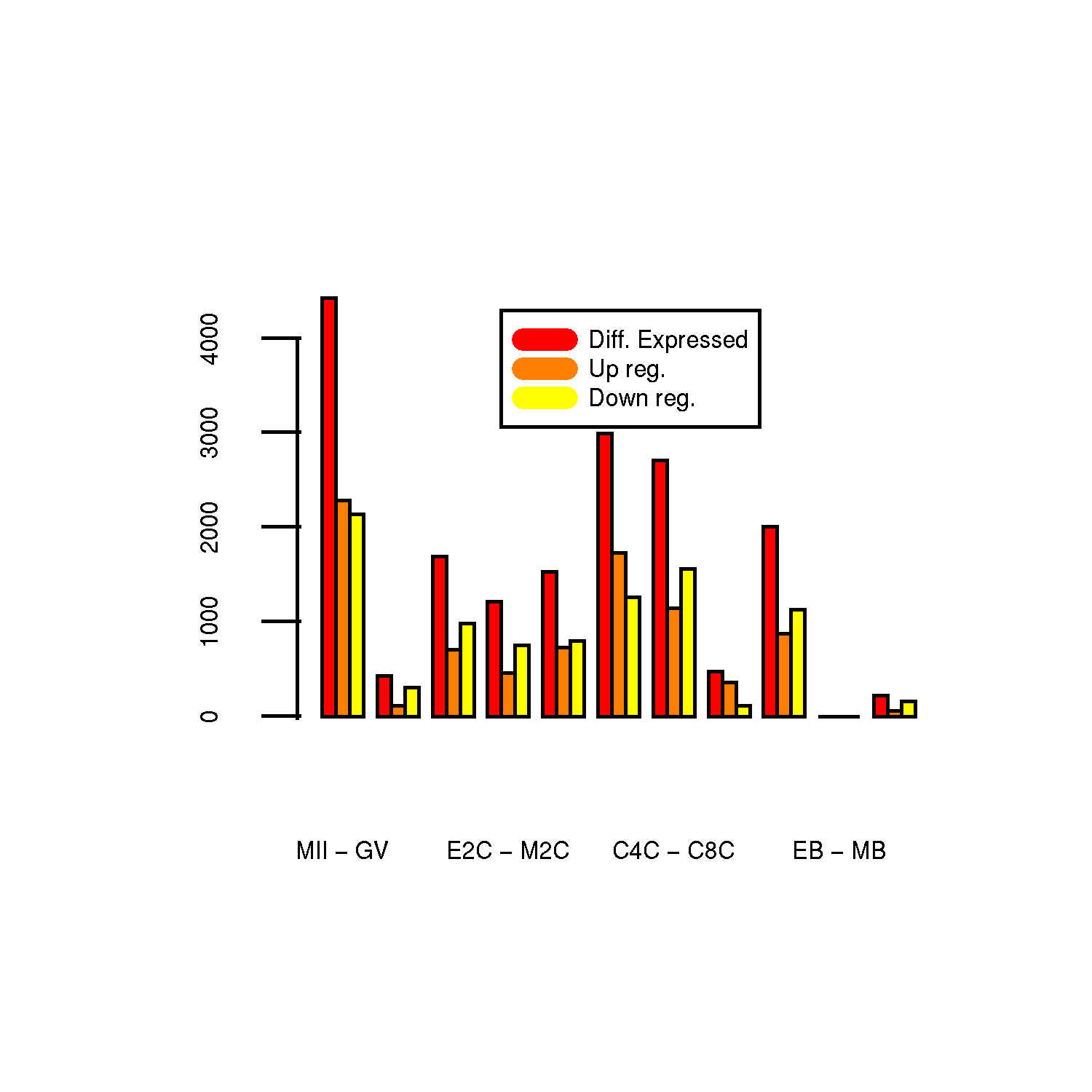

Supplement: Figure S7 — Differential expression in mice. Yellow bars represent the number of probe sets with lower expression, the orange those with higher expression and the red ones the sum of the two. (6.75 MB TIF) [file pone.0007844.s026.tif]
